# RedOx regulation of LRRK2 kinase activity by active site cysteines

**DOI:** 10.1038/s41531-024-00683-5

**Published:** 2024-04-03

**Authors:** Chiara R. Trilling, Jui-Hung Weng, Pallavi Kaila Sharma, Viktoria Nolte, Jian Wu, Wen Ma, Daniela Boassa, Susan S. Taylor, Friedrich W. Herberg

**Affiliations:** 1 Department of Biochemistry, University of Kassel, Kassel, Germany; 2 Department of Pharmacology, University of California, San Diego, CA USA; 3 Department of Physics, University of Vermont, Burlington, VT USA; 4 National Center for Microscopy and Imaging Research, University of California, San Diego, CA USA; 5 Department of Neurosciences, University of California, San Diego, CA USA; 6 Department of Chemistry and Biochemistry, University of California, San Diego, CA USA

**Keywords:** Proteins, Computational biology and bioinformatics, Parkinson's disease

## Abstract

Mutations of the human leucine-rich repeat kinase 2 (LRRK2) have been associated with both, idiopathic and familial Parkinson’s disease (PD). Most of these pathogenic mutations are located in the kinase domain (KD) or GTPase domain of LRRK2. In this study we describe a mechanism in which protein kinase activity can be modulated by reversible oxidation or reduction, involving a unique pair of adjacent cysteines, the “CC” motif. Among all human protein kinases, only LRRK2 contains this “CC” motif (C2024 and C2025) in the Activation Segment (AS) of the kinase domain. In an approach combining site-directed mutagenesis, biochemical analyses, cell-based assays, and Gaussian accelerated Molecular Dynamics (GaMD) simulations we could attribute a role for each of those cysteines. We employed reducing and oxidizing agents with potential clinical relevance to investigate effects on kinase activity and microtubule docking. We find that each cysteine gives a distinct contribution: the first cysteine, C2024, is essential for LRRK2 protein kinase activity, while the adjacent cysteine, C2025, contributes significantly to redox sensitivity. Implementing thiolates (R-S^-^) in GaMD simulations allowed us to analyse how each of the cysteines in the “CC” motif interacts with its surrounding residues depending on its oxidation state. From our studies we conclude that oxidizing agents can downregulate kinase activity of hyperactive LRRK2 PD mutations and may provide promising tools for therapeutic strategies.

## Introduction

The leucine-rich repeat kinase 2 (LRRK2) is a large multi-domain protein that belongs to the family of tyrosine kinases-like (TKL) protein kinases^1^, and mutations of the LRRK2 gene have been identified as one of the genetic causes of Parkinson’s disease (PD), the second most common progressive neurodegenerative disorder^2,3^. Its enzymatic core contains a kinase domain (KD) and a GTPase domain (ROC), thereby combining two important biological switches for cellular regulation and signal transduction in a single polypeptide chain. Most of the PD-associated mutations are located within or adjacent to these enzymatic domains, with G2019S and I2020T in the kinase domain, R1441C/H/G in the ROC domain^4–7^ and Y1699C at the interface of the COR-B and ROC domain^7,8^. Kinase activity-modulating mutations are associated with PD^9^, with G2019S as the most common pathogenic mutation that leads to increased kinase activity^10–12^. Although several mechanisms for LRRK2 regulation have been elucidated, which include auto-phosphorylation^13,14^, protein-protein interactions (e.g., 14–3–3 protein binding)^15–17^ or allosteric intradomain crosstalk^8^, the intricate mechanism of LRRK2 kinase regulation is still elusive.

A major regulatory mechanism for eucaryotic protein kinases (ePKs) is reversible Ser/Thr post-translational phosphorylation in the Activation Segment (AS, aa2017–2042) that extends from the conserved DFG motif to the APE motif. In LRRK2, the phenylalanine of the DFG motif is replaced by a tyrosine (Y2018), resulting in a DYGψ motif, which serves as a “brake” for catalytic activity^18^. While LRRK2 contains three putative phosphorylation sites (T2031, S2032, T2035) in the AS, none of those have been correlated with activation. However, other unrevealed, distinct regulatory mechanisms may be embedded in the AS. In addition to the conserved Activation Loop (AL, aa2021–2031) phosphorylation, redox regulation is a commonly reported mechanism for regulation of both protein phosphatases and protein kinases. The oxidation of a critical cysteine residue that can reversibly inhibit protein tyrosine phosphatases (PTP) was first reported in 2003^19^, and this launched a new era in redox regulation of signaling^20^. In the early 1980s, a redox-sensitive cysteine, C199, was identified in the AS of protein kinase A (PKA)^21–23^. Since then, several examples of redox-based modulation of catalytic activity have been reported for both, Ser/Thr protein kinases, including PKG or Aurora A^24–26^ and Tyr protein kinases (e.g., SCR or EGFR)^27,28^. We already proposed earlier the involvement of two adjacent cysteine residues in the LRRK2 AL potentially involved in redox-regulation^18^.

Cysteine residues play important roles in redox mechanisms acting via highly reactive sulfur-containing thiol (-SH) groups, which can exist in several oxidation states (ranging from –2 to +6)^29^. Oxidation of a free thiol group results in the formation of a transient sulfenic acid species (-SOH), enabling S-nitrosylation, S-glutathionylation or reaction with an additional free thiol group leading to the formation of a disulfide bond^29,30^. Further oxidation to sulfinic acid (-SO_2_H), followed by a sulfonic acid (-SO_3_H), results in an irreversible modification. The high reactivity of cysteines results in various regulatory effects, including inhibition or activation upon oxidation or disulfide bond formation. This underpins the importance of investigating the complexity of the “Cysteinome” and understanding how redox-based regulation contributes to the rapidly growing research area of drug development based on cysteine-containing proteins.

LRRK2 is unique as it is the only protein kinase with two adjacent cysteines in its Activation Loop (AL, aa2021–2031). In this study, we investigated the role of this “CC” motif, which allows to permit redox sensitivity for LRRK2 kinase activity. We propose a unique mechanism of cysteine-based regulation in LRRK2, distinct from previously described evolutionarily conserved regulatory mechanisms found in many Ser/Thr protein kinases. Employing an approach combining site-directed mutagenesis with biochemical analysis, cell-based assays, and Gaussian accelerated Molecular Dynamics (GaMD) simulations, we show that LRRK2 kinase activity can be reversibly regulated by oxidizing and reducing agents. We attribute the distinct role of both AS cysteine residues in controlling kinase activity using peptides or protein substrates and by studying LRRK2 autophosphorylation. We also demonstrate that microtubule association of LRRK2 is redox-dependent. Finally, in an attempt to mimic the reactive state of C2024 and C2025, we embedded thiolates (R-S^-^) in the GaMD simulations and propose a model, where dual cysteine modification in the AS allows finetuning of LRRK2 kinase activity.

## Results

**Fig. 1:**
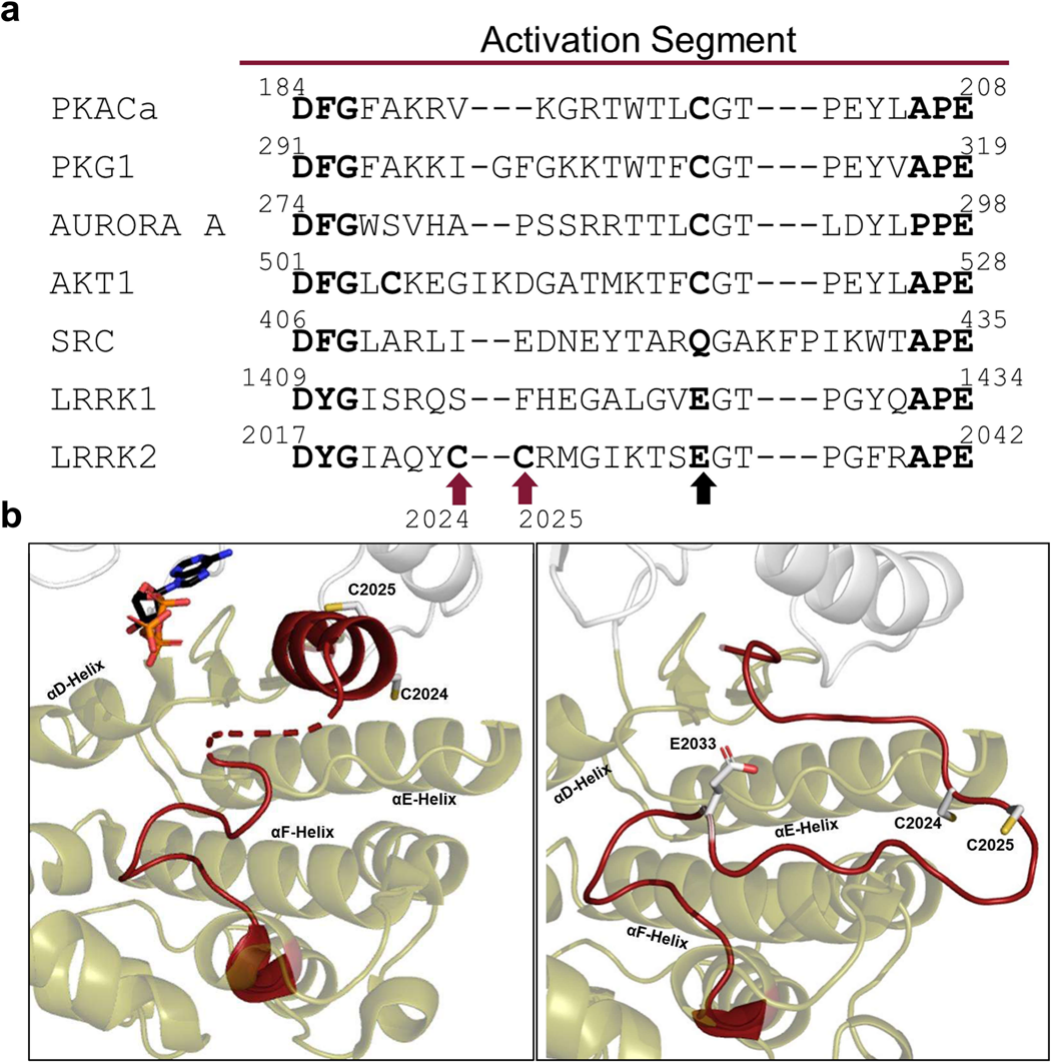
Two adjacent cysteines in the LRRK2 Activation Segment. **a** A sequence alignment of selected human protein kinases revealed two unique cysteines, C2024 and C2025 (red arrows), in the Activation Segment (AS) of LRRK2. The highly conserved region between the DFG/DYGψ and APE motif (bold) of PKACα, PKG1, AURORA A, AKT1, SRC, LRRK1 and LRRK2 was aligned using CLUSTAL O (1.2.4.). Cys199 in PKA is conserved in many S/T-kinases, but not in SRC, LRRK1 and LRRK2, where it is a glutamic acid (black arrow). **b** In inactive FL LRRK2 (left, PDB: 7LHW) C2024 and C2025, are located within the helical motif formed by the AS (red). C2025 was directed towards the active site, while C2024 pointed in the opposite direction. The predicted active conformation of LRRK2 (right, AlphaFold2), showed repositioning of C2024 towards surrounding domains.

Many eukaryotic protein kinases (ePKs) are known to be regulated in a redox-sensitive manner, and particularly in the Activation Segment (AS), canonical sequences embedding a critical cysteine residue have been identified. A sequence alignment of the AS of selected human protein kinases revealed that in contrast to AGC kinase family members (PKA, PKG1, Aurora A, and AKT1), the canonical cysteine residue (corresponding to C199 in PKA) is not conserved in LRRK2 as a member of the TKL family, where it is a glutamic acid (E2033) (Fig. 1a). Also, in SRC, a member of the Tyrosine Kinase (TK) family, this canonical cysteine is replaced by glutamine. Interestingly, LRRK2 contains two adjacent, highly unique cysteines in the AS, C2024 and C2025. So far, several protein kinases are known to have at least one cysteine at the described positions in LRRK2 (LRRK2_2024_: CLIK1L, TSSK1, TSSK2; LRRK2_2025_: VRK1, VRK2, DYRK4, JNK2, ERK5), but no other kinases have two adjacent cysteines at these positions.

### Two adjacent Cysteine residues change orientation in the LRRK2 kinase domain upon activation

In the full-length (FL) LRRK2, cryo-EM structure (PDB: 7LHW, Supplementary Fig. 1), the kinase domain (KD) is surrounded by the N-terminal and C-terminal domains (NtDs and CtDs), locking the kinase in its inactive conformation^31^. Various characteristics of an inactive kinase are combined in this FL LRRK2 structure. The highly conserved regulatory (R)-Spine is broken^32^, the αC-helix is flipped out, and the Y2018 from the DYGψ motif prevents interaction of E1920 and K1906 with ATP^33^. Remarkably, the first ten amino acids of the AS (aa2017–2026), including the DYGψ motif, form a very stable helix^31,33^, which additionally prevents the kinase from being in an active state. The two adjacent cysteines, C2024 and C2025, are part of this helical motif (Fig. 1b), whereby C2024 in the inactive FL cryo-EM structure is oriented towards the active site cleft, while C2025 points in the opposite direction and is solvent exposed (Fig. 1b left). However, in an active structure of FL LRRK2, predicted by AlphaFold2, the helical motif in the AS is unfolded, and the C2024 and C2025 are reorientated allowing the AL to approach the surrounding COR-B and ROC domains (Fig. 1b right). Additionally, E2033, replacing in LRRK2 the canonical cysteine, is not resolved in the inactive FL cryo-EM structure, while in the active state, predicted by AlphaFold2, E2033 interacts with T2031, one of the putative phospho-sites in the AS (Fig. 1b left).

### C2024 and C2025 are critical for redox regulation of LRRK2 kinase activity

**Fig. 2:**
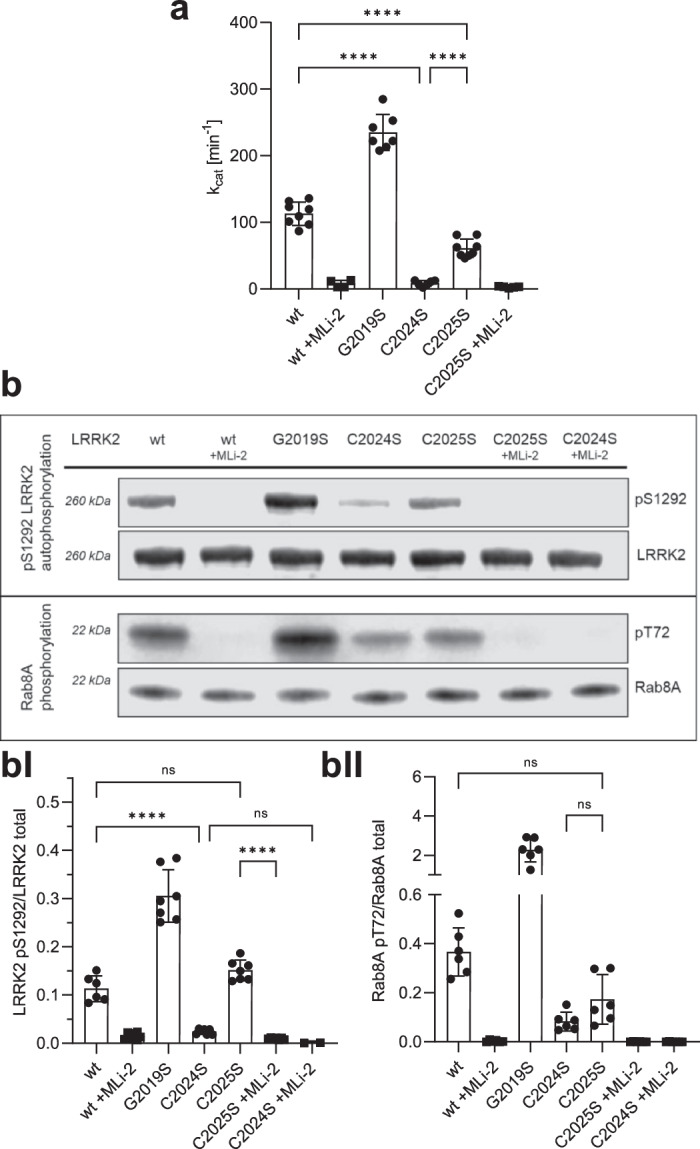
In vitro characterisation of C2024S and C2025S LRRK2 mutants. **a** LRRK2 kinase activity was determined using LRRKtide as a peptide substrate employing a microfluidic mobility shift assay (MMSA). Kinase activity of C2024S was abolished and activity of C2025S was decreased in comparison to LRKK2 wt. **b** LRRK2 kinase activity was measured either by auto-phosphorylation on S1292 using specific antibodies against pS1292-LRRK2 (Abcam MJFR-19–7–8; upper blot, quantification **bI**) or by Rab8A phosphorylation on T72 (Abcam MJF-R20; lower blot, quantification **bII**). Both kinase assay showed reduced activity for C2024S, while phosphorylation of C2025S was comparable to LRRK2 wt. The type I kinase inhibitor MLi-2 was used as a control in all assays. Representative Blots are shown, and data points represent the standard deviation (SD) of at least two protein preparations with three independent measurements based on a one-way ANOVA with a multiple comparison n.s.: *P* ≥ 0.05; **: *P* < 0.01; ***: *P* < 0.001; ****:*P* < 0.0001.

To investigate the function of C2024 and C2025 in LRRK2 kinase regulation, we generated LRRK2 Cys-to-Ser substitutions at both positions, whereby serine was chosen as a non-redox sensitive cysteine mimic because of its similar size, and structure. The hyperactive, pathogenic LRRK2 G2019S mutant was used as a control and kinase activity for LRRK2 wt, and mutants were tested in the absence and presence of the highly specific ATP-competitive (type I) LRRK2 kinase inhibitor, MLi-2^34^. Using a microfluidic mobility shift assay (MMSA) with LRRKtide (RLGRDKYKTLRQIRQ) as a peptide substrate, we showed that the C2024S substitution completely abolished kinase activity (Fig. 2a). A commonly used substitution with alanine introduced into C2024 again yielding a kinase inactive protein (Supplementary Fig. 2). In contrast, kinase activity of the C2025S mutant protein was still detectable, however, significantly decreased compared to LRRK2 wt and further inhibited by MLi-2, indicating that the mutant protein can still bind ATP as well as MLi-2. We also tested the ability of LRRK2 wt and the mutant proteins to be auto-phosphorylated on S1292 - a commonly used read-out to analyse LRRK2 kinase activity. Although in MMSA no kinase activity could be detected for C2024S (Fig. 2a), auto-phosphorylation of pS1292 in C2024S protein was dramatically reduced, but still detectable (Fig. 2b, BI), and LRRK2 C2025S protein showed pS1292 auto-phosphorylation comparable to LRRK2 wt (Fig. 2b, BI). For both mutant proteins, C2024S and C2025S, treatment with MLi-2 fully abolished phosphorylation of S1292. In addition, we assayed for trans-phosphorylation of a heterologous substrate, Rab8A on T72, which was significantly decreased for both, LRRK2 C2024S and C2025S compared to LRRK2 wt and again was abolished by using MLi-2 (Fig. 2b, BII). These data indicate the importance of both cysteines within the AS to modulate LRRK2 kinase activity, although both residues may be involved in regulation differently. C2024, in particular, appears to play an important role in a regulatory mechanism of catalytic activity that has not yet been described for LRRK2.

### Mutation of C2024 resembles a kinase-dead phenotype in a MT docking assay

We then used a cell-based assay to evaluate the capacity of LRRK2 wild-type and cysteine mutations to form filaments that correlate with docking onto microtubules (MT). It has been previously shown that some of the familial PD-mutations of LRRK2 dock spontaneously onto microtubules^35–38^.

**Fig. 3:**
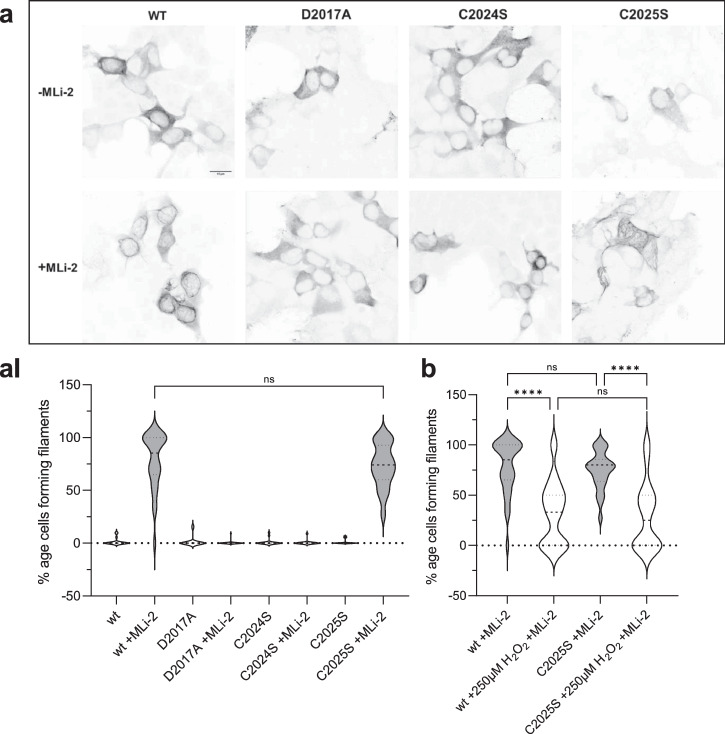
LRRK2 docking unto microtubules is influenced by oxidation of C2024. **a** A filament formation assay was performed in HEK293T cells overexpressing LRRK2 constructs 48 h after transfection. LRRK2 wt in the presence of MLi-2 (100 nM) showed filament formation (80%, **aI**) of the transfected cells. In the absence of MLi-2 LRRK2 were cytosolically distributed. The kinase dead mutant D2017A was used as a control and is cytosolically distributed in the absence and presence of MLi-2. The C2024S mutant also showed the kinase dead phenotype while the C2025S mutant behaved like the wt (**a, aI**). **b** LRRK2 wt and C2025S showed significantly reduced filament formation in the presence and absence of 250 μM H_2_O_2_. Representative images, scale bar: 15 μm; (**aI**, **b**). Percentages of cells exhibiting LRRK2 filament formation in the absence and presence of MLi-2. Data are shown as violine plots (median and upper and lower quartiles) from three independent sets of experiments based one-way ANOVA with with a multiple comparison n.s.: *P* ≥ 0.05; **: *P* < 0.01; ***: *P* < 0.001; ****:*P* < 0.0001.

HEK293T cells were transiently transfected with Flag-tagged LRRK2 full-length, wild-type and mutants, and the ability of overexpressed LRRK2 wild-type and mutant proteins to dock onto microtubules in the absence and presence of MLi-2 was assessed as previously well described^35^. C2024S behaved similar to the D2017A kinase-dead mutant; it showed no docking onto microtubules even in the presence of MLi-2 (Fig. 3a, AI). Both, the C2024S and the D2017A mutations can render the kinase domain inactive and suggest that an inactive open conformation of the kinase domain prevents docking to MTs. This inactive open conformation was captured by modeling a type II inhibitor^39^. In contrast, C2025S behaved like wt LRRK2; it could dock onto microtubules only in the presence of MLi-2 in ~75% of the cells (Fig. 3a, AI). Upon treatment of the transfected cells with 250 μM H_2_O_2_, the ability of wt and C2025S to dock onto microtubules was reduced by over twofold (Fig. 3c). These results are in line with the in vitro experiments.

### Reducing and oxidizing agents reversibly modulate LRRK2 kinase activity

Oxidation processes are a common mechanism for regulating the catalytic activity of protein kinases^40,41^. Because loss of catalytic activity is often due to oxidation of critical sulfur-containing amino acids like cysteines and methionines, reductants like DTT (dithiothreitol) or TCEP (tris(2-carboxyethyl)phosphine) are commonly used in biochemical in vitro kinase assays. To elucidate the potential importance of C2024 and C2025 as redox-sensitive cysteines, we thus asked how different oxidants and reductants affect kinase activity of LRRK2 wt, the pathogenic G2019S mutant, and the C2025S mutant proteins. LRRK2 wt and mutant proteins were treated either with DTT (reductants) or different oxidants (hydrogen peroxide (H_2_O_2_), NCA (1-nitrosocyclohexalycetate) or CXL-1020), followed by determination of kinase activity using LRRKtide in a MMSA. NCA and CXL-1020 are two Nitroxyl-(HNO) donors^42^, which target selectively cysteinyl thiols due to their electrophilic properties, resulting in covalent modifications of cysteines or formation of disulfide bonds.

**Fig. 4:**
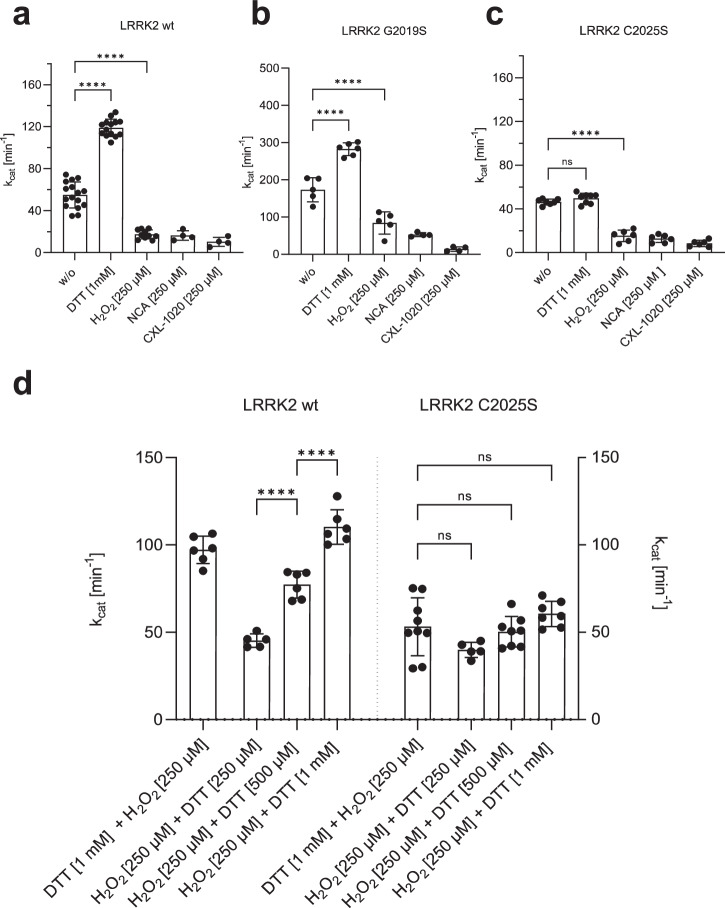
Redox-dependent regulation of LRRK2. **a–c** Increased kinase activity towards LRRKtide was determined for both, LRRK2 wt **a** and G2019S **b**, after reduction with 1 mM DTT, while activity of C2025S **c** was unaffected. Oxidation with 250 µM H_2_O_2_, NCA or CXL-1020 resulted in reduced activity of LRRK2 wt, G2019S and C2025S. **d** An experimental setup was approached to test if reversible oxidation of controls LRRK2 kinase activity. First LRRK2 wt and C2025S were preincubated for 20 min with either H_2_O_2_ (250 µM) or DTT (1 mM), followed by incubation for 20 min with DTT (1 mM) or H_2_O_2_ (250 µM, 500 µM, 1 mM). After oxidation with 250 µM H_2_O_2_, LRRK2 wt kinase activity was reactivated with DTT in a concentration dependent manner. C2025S showed reduced activity even in the presence of DTT and abolished reactivation with DTT. Data points represent the standard deviation (SD) of at least two protein preparations with three independent measurements based on a one-way ANOVA with a multiple comparison n.s.: *P* ≥ 0.05; **: *P* < 0.01; ***: *P* < 0.001; ****:*P* < 0.0001.

Recombinant LRRK2 wt and mutant proteins were purified and stored under slightly reducing conditions in the presence of 0.5 mM TCEP (see M&M). Increasing concentrations of DTT were test (Supplementary Fig. 3) and adding 1 mM DTT resulted in a 2.0-fold increase in LRRK2 wt kinase activity, whereas oxidation with H_2_O_2_, NCA, and CXL-1020 (250 µM each) significantly inhibited LRRK2 kinase activity in the MMSA (Fig. 4a). The activity of LRRK2 G20219S was also increased in the presence of 1 mM DTT (1.6-fold) (Fig. 4b), and oxidation with all three oxidizing agents significantly inhibited kinase activity. Interestingly, CXL-1020 showed the strongest inhibition on the hyperactive mutant (G2019S, Fig. 4b). LRRK2 C2025S protein showed no activation by reduction with DTT, while oxidation with all three oxidants resulted in inhibition of kinase activity (Fig. 4c). These findings suggest that the ability of LRRK2 to phosphorylate peptide substrates is inhibited by oxidation. Our results suggest that C2024 is unusually reactive and essential for kinase activity. In contrast, C2025, while not essential, may also play a role in modulating redox sensitivity. Only when both cysteines are present maximum activity can be achieved, reflected in conditions using 1 mM DTT. Surprisingly, even though LRRK2 contains a total of 64 cysteines, mutation of a single cysteine (C2024) in the AS essentially abolishes kinase activity, while modification of C2025 significantly alters the activity.

Having established that oxidation and reduction have a considerable impact on LRRK2 kinase activity, we asked if oxidation is reversible. LRRK2 wt and C2025S mutant proteins were preincubated for 20 min either with 250 µM H_2_O_2_ or 1 mM DTT, respectively, followed by an incubation for 20 min with 250 µM, 500 µM and 1 mM DTT or 250 µM H_2_O_2_. As shown in Fig. 4d, reduction of LRRK2 wt with DTT is reversible following treatment with H_2_O_2_. Adding hydrogen peroxide initially results in slightly decreased kinase activity while treatment with increasing doses of DTT show that oxidation is reversible in a dose-dependent manner (Fig. 4d), as reflected in an DTT-dependent increase in LRRK2 wt kinase activity. For LRRK2 C2025S mutant protein, no effect of either oxidant (H_2_O_2_), after previous reduction, or reducing agent could be detected (Fig. 4d). Together, these data indicate that LRRK2 catalytic activity follows a so far undescribed redox-based regulatory mechanism, allowing a switch between an oxidized, “inactive” and a reduced “active” state.

### Assessing the role of adjacent cysteines in the Activation Loop with MD simulations

**Fig. 5:**
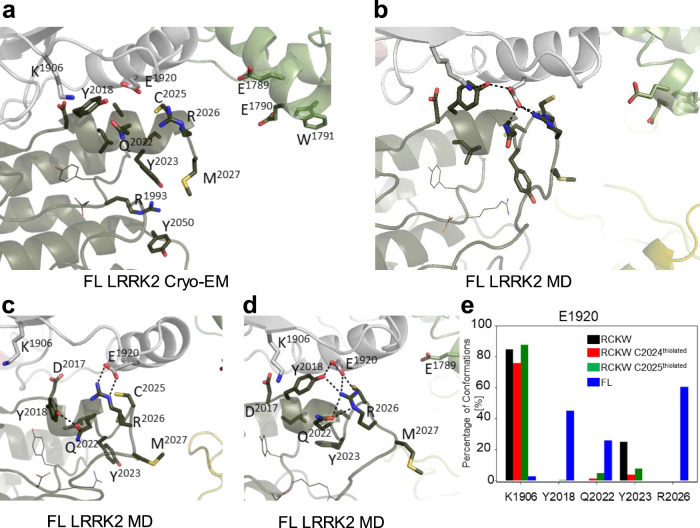
MD simulation revealed interactions stabilizing the inactive state of the Activation Loop in FL LRRK2. **a** The cryo-EM structure of inactive full-length (FL) LRRK2 (PDB: 7LHW). The Activation Loop (aa2021–2031) is ordered except for three disordered residues (aa2028-2030). The DY_2018_G motif through R2026 forms a stable helix that would inhibit kinase activity. **b** Simulation of FL LRRK2 based on the cryo-EM structure, showing the inhibitory helix (aa2017-2026) and P + 1 loop are stable. **c, d** Two snapshots that capture different of AL residues with active site residues. **e** The bar graph quantitates transient interactions of E1920 with K1906, Y2018, Q2022, Y2023 and R2026 during simulations. Simulations were performed with both C2024 and C2025 in the thiolated and non-thiolated form using the RCKW cryo-EM structure (PDB: 6VNO). B MD simulation revealed interactions stabilizing the inactive state of the Activation Loop in FL LRRK2.

Given that the biochemical data supports the importance of C2024 as a reactive cysteine in the AL of LRRK2, we sought to mimic the reactive cysteines computationally. Specifically, we asked if the GaMD simulations would be influenced if C2024 or C2025 were deprotonated. The protonation states of two adjacent histidine residues have been found to be important for the functions of various proteins such as photolyase enzyme (PHR)^43^, human prion protein (PrP)^44^, and human forkhead box protein P1 (FoxP1)^45^. In the FL cryo-EM inactive LRRK2 structure only three residues (aa2028–2030) are disordered^33^ and the inhibitory helix in the AL locks the kinase into an inactive state. The DYGψ motif is part of the inhibitory helix, and, based on our earlier GaMD simulations, this helix is very stable (Fig. 5a)^31,33^. This inhibited conformation remains stable during the simulations (Figure/Video 5B). When the kinase assumes an active conformation, the regulatory triad (E1920, K1906 and D2017) needs to be in place^35,46^. Specifically, E1920 in the αC-helix should interact with K1906 in β3 sheet when LRRK2 is active. In addition, D2017 in the DYGψ motif in the C-Lobe should also be close to K1906 in the N-Lobe. In the FL LRRK2 cryo-EM structure, E1920 is far from K1906; however, it is close to Y2018 in the DYGψ motif (Fig. 5c, d), which is not consistent with an active kinase conformation. In contrast, in the cryo-EM structure, R2026 in the AL is solvent exposed and far from E1920. Based on the MD simulations, however, R2026 is interacting tightly (~ 50%) with E1920 in the αC-helix, which contributes to keeping the αC-helix of the N-Lobe in an “out” conformation (Fig. 5e). The simulations also confirmed that E1920 rarely interacts with K1906. In addition, the simulation showed even stronger interaction of the Y2018 hydroxyl with E1920 (~60%). This stable inhibited conformation consisting of Y2018, E1920 and R2026 is similar to what is seen in the inactive conformation of SRC where the DFG motif is in an inactive helical conformation and a basic residue in the AL is interacting with the conserved glutamate in the αC-helix^47^. In this inhibited state the AL of LRRK2 is constrained and does not approach the COR-B:ROC interface when either C2024 or C2025 is thiolated (R-S^-^).

**Fig. 6:**
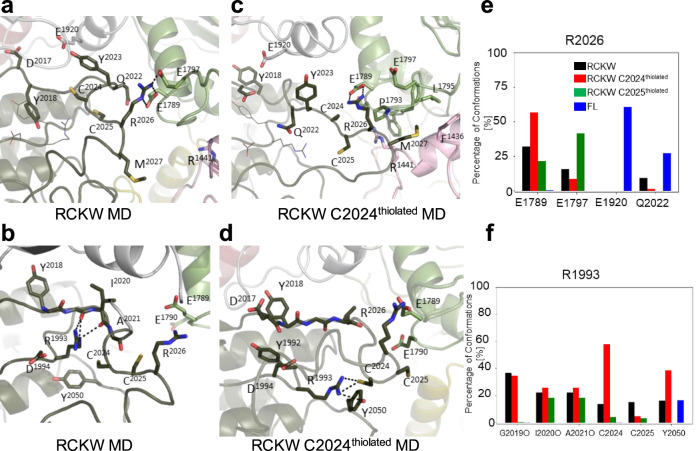
MD simulations showed the interactions of R2026 in the Activation Loop and R1993 in the HRD motif. In the RCKW cryo-EM structure (PDB: 6VNO), the Activation Loop (aa2021-2031) was flexible and was thus missing in the model. For the simulations we modeled in the missing residues. **a–d** Snapshots from MD simulations of this region are shown. For the simulation, we compared FL LRRK2 as well as the RCKW structure with two versions of the RCKW where either C2024 or C2025 were thiolated. **a** In the RCKW R2026 on the activation loop interacted with E1789 and E1797 in the COR-B domain, **b** R1993 in the HRD motif interacts with the backbone of G2019 and I2020 in RCKW. **c** In the simulation of C2024^thiolated^, the R2026-E1789 interaction increased. **d** R1993 interacts with C2024 and Y2050. **e** Bar graph illustrating R2026 interactions seen in the simulations. No interactions were observed between R2026 and COR-B in the inactive FL LRRK2. **f** Bar graph illustrating R2023 interactions seen in the simulations: Backbone (G2019, I2020, A2021) and sidechain (C2024, C2025, Y2050).

In contrast to full-length LRRK2, in the cryo-EM LRRK2^RCKW^ structure^48^ the inhibitory helix is broken, and the AL (aa2020–2031) is mostly disordered. GaMD simulations of this structure show that the AL does come much closer to the interface between the COR-B helix and the ROC domain where many PD mutations are located. Here, we looked more carefully at the effects of introducing a negative charge on C2024 and C2025. As seen in Fig. 5e, in all the LRRK2^RCKW^ simulations, K1906 is interacting strongly with E1920, which is typical of an active kinase. In this conformation, however, R2026 in the AL now interacts with the COR-B helix, specifically with E1789, which is in close proximity to W1791, a key residue at this interface that interacts with the side chain of the known PD mutation site R1441 (Fig. 6a). In FL LRRK2, R2026 never samples E1789. Surprisingly the frequency of this interaction is also enhanced when C2024 is thiolated (Fig. 6c). In active kinases R1993 in the HRD motif typically interacts with phosphorylated residues in the AL^49^ (Fig. 6b). We thus asked if the space that R1993 explores is changed when either of the AL cysteines is thiolated. As seen in Fig. 6d, the interaction of R1993 with C2024 is significantly increased when C2024 is thiolated. This is in contrast to the inactive FL LRRK2 where the side chain of R1993 samples many negative charged sites^33^. The interaction of R2026 with E1797, another residue that is at the ROC:CORB interface, is reduced when C2024 is thiolated but increased when C2025 is thiolated (Fig. 6e). Another key residue that lies between the APE motif and the conserved aspartate (D2055) at the beginning of the αF-helix is Y2050. In fully active kinases with a phosphorylated AL, this side chain is anchored to the HRD arginine (R1993). The interaction of the sidechain of Y2050 with R1993 is also significantly increased when C2024 is thiolated (Fig. 6f). In all other structures so far, including in the cryo-EM of FL LRRK2 (Fig. 5a) Y2050 is pointing away from the HRD arginine.

**Fig. 7:**
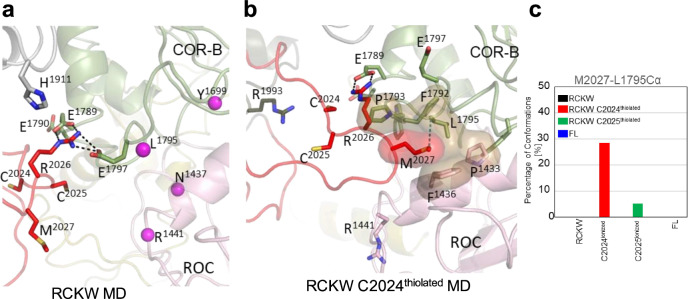
MD simulations capture the interactions of R2026 and M2027 in the Activation Loop with the COR-B/ROC interface. The space between COR-B and the ROC domain is the site of many PD mutations (R1441, N1437, Y1699 and L1795, shown in sphere). **a** Interactions of R2026 with E1789 and E1797 are captured in the simulations. **b** A major interaction of M2027 dock into the interface between COR-B/ROC domain is captured when C2024 is thiolated. M2027 is surrounded by hydrophobic residues. **c** The bar graph illustrates the interaction between Sulfur of M2027 and Cα of L1795 interactions during simulations. The interface shown in **b** is never explored in the RCKW and FL LRRK2 structures.

In the LRRK2^RCKW^ simulation, the AL samples space closer to COR-B. As indicated above, R2026 within the AL interacts with both, E1789 and E1797 in COR-B (Fig. 7a). When C2024 is thiolated, the regions beyond R2026 also approach the COR-B:ROC interface more closely. Specifically, M2027 is sometimes found in the space between these two domains (Fig. 7b), whereas in FL LRRK2 (Fig. 5a) this methionine is interacting with other hydrophobic residues in the AS of the kinase domain. When M2027 docks into the interface between the COR-B and ROC domains, it is surrounded by a hydrophobic shell comprised of residues: P1433 and F1436 from the ROC domain, and F1792, P1793, and L1795 from the COR-B domain. M2027 explores this hydrophobic space less when C2025 is thiolated; however, the docking of M2027 into this hydrophobic space is never observed in simulations of the full-length inactive LRRK2 or in the LRRK2^RCKW^ construct (Fig. 7c).

## Discussion

This study demonstrates that two adjacent redox-sensitive cysteines, the “CC” motif, in the Activation Segment (AS, aa2017–2042) effectively controls LRRK2 kinase activity. A single substitution of each cysteine has a major impact on LRRK2 kinase activity. Substitution of C2024, at a position which is five amino acids downstream the conserved DYGψ motif (DYGψ + 5), with serine abolishes phosphorylation not only of the peptide substrate LRRKtide (RLGRDKYKTLRQIRQ), but also of the physiological substrate Rab8A as well as auto-phosphorylation on S1292. C2024A also abolished kinase activity in the MMSA (Supplementary Fig. 2). The adjacent cysteine, C2025, at the DYGψ + 6 position, is highly sensitive to oxidation and essential for full LRRK2 kinase activity. When C2025 is replaced with serine, the activity is reduced but not abolished. Expression levels of the C2024S/C2025S double mutant in HEK293T cells were too low for both protein purification and MT docking assays.

Cysteine residues are key players in redox-based protein regulation. Although such a mechanism has not been reported for LRRK2, redox-dependent regulation has been described for several protein kinases, including members of the AGC kinase family and the Tyrosine kinase (TK) family. Although the mechanisms for redox regulation likely differ for each kinase, our data suggests that redox regulation of the AL is also critically important for LRRK2.

Reversible AL-phosphorylation is another mechanism of regulation in ePKs and has already been investigated in the context of AL-cysteine modification^24,50^. It was demonstrated that oxidation of C199 in the AL of PKA inactivates the kinase activity^51^ while replacement of C199 with alanine does not inhibit activity but does facilitate dephosphorylation of pT197, which in turn is crucial for kinase activity^23,51,52^. A similar mechanism has been proposed for Aurora A, another member of the AGC kinase family; again oxidation of the canonical C290 in the AL inhibits its catalytic activity^24^. Even though the canonical C199 (PKA) and C290 (Aurora A) effect kinase activity in different ways upon oxidation, bioinformatics analysis revealed that these cysteine residues at the phospho-site+2 position in the AS are conserved (~11.5%) in human Ser/Thr protein kinases^24^. Taken together, redox modulation of conserved cysteines adjacent to the phospho-site within the AS of human protein kinases adds an important additional layer to the conserved phosphorylation-dependent regulation mechanism, which allows fine-tunning of protein kinase activity^24,51,53^.

This situation is quite different and unique for LRRK2, where a sequence alignment showed that this cysteine at the phospho-site+2 (P-site) position is not conserved; it is replaced with glutamate (E2033). This is also true for LRRK1. Furthermore, of any human protein kinase only LRRK2 contains the described adjacent cysteines in the AL. Both, the lack of the conserved canonical cysteine in the P + 2 position and the presence of the regulatory “CC” motif in the AL indicates that redox-based regulation of LRRK2 follows a unique mechanism distinct from previously described redox mechanisms.

Moreover, LRRK2 is one of a few examples in the human kinome with a DYGψ motif, instead of the highly conserved DFG motif. Replacement of Y2018 with phenylalanine results in a hyperactive LRRK2 mutant, with higher kinase activity than the pathogenic G2019S mutant^18^. Schmidt et al. revealed that Y2018 is a critical residue for stabilizing the inactive conformation of LRRK2 due its hydroxyl group, therefore in the Y2018F mutant, precise regulation of the R-spine assembly is influenced^18^. Also, in PKA mutation of the F185 to a tyrosine (F185Y) corresponding to Y2018 in LRRK2 increased its catalytic activity^18^. Besides LRRK2 the only serine/threonine kinases that have a tyrosine instead of the phenylalanine in the DFG motif are LRRK1, VRK1 and NEK9 have^18^. However, VRK1 contains a cysteine residue (C205) corresponding to C2025 in LRRK2, but so far, little is known about redox sensitivity of VRK1.

Since oxidation of proteins often results in loss or modulation of function, reducing agents like DTT (dithiothreitol), TCEP (tris(2-carboxyethyl) phosphin-hydrochlorid) or β-mercaptoethanol are commonly used in in vitro kinase studies to prevent oxidation of key cysteine residues. Here, LRRK2 is purified and stored in 0.5 mM TCEP to maintain structural and catalytic properties. Remarkably, further reduction of LRRK2 wt and G2019S with DTT increased catalytic activity, while the LRRK2 C2025S mutant protein was unaffected by adding additional reducing agents. These findings suggest that C2025 contributes to redox sensitivity, and reduction of this specific cysteine residue allows for full kinase activity. LRRK2 is also susceptible to reversible oxidation by H_2_O_2_ or by the HNO-related oxidants NCA and CXL-1020, developed as prodrugs targeting the cGMP-dependent protein (PKGIα) kinase for treatment of cardiovascular diseases^25,54,55^. From these results we hypothesize that in the C2025S mutant protein further oxidation and subsequent inhibition of LRRK2 kinase activity is due to oxidation of C2024 which still carries a reactive thiol group even though reduction of C2025S mutant protein does not result in an increase in catalytic activity. This means that for full kinase activity both thiol groups of C2024 and C2025 must be present, however, the two cysteines most likely contribute differently.

The effect of H_2_O_2_ on LRRK2 in HEK293 cells was previously investigated by Di Maio et al. ^56^. They demonstrated an H_2_O_2_ dose-dependent activation of LRRK2 kinase activity by measuring pS1292 as well as Rab10 phosphorylation using a proximity ligation (PL) assay. This contrasts with our in vitro experiments where for purified recombinant LRRK2 in the presence of ATP-Mg and GDP we observed a clear inhibition by oxidizing agents including H_2_O_2_. In addition, we used a cell-based assay in HEK293T cells, where LRRK2 can dock onto MTs. While many PD mutants dock spontaneously in a putative active conformation of the kinase domain onto MTs^38,39^ for LRRK2 wt docking requires the presence of the type I inhibitor MLi-2^18^. Neither C2024S or C2025S dock spontaneously onto MTs and only C2025S forms filaments in the presence of MLi-2, similar to LRRK2 wt and G2019S. In contrast, for LRRK2 C2024S no filament formation was observed either in the presence or absence of MLi-2 indicating that the LRRK2 C2024S is most likely in an inactive and open conformation similar to the kinase-dead D2017A mutant. Following oxidation by H_2_O_2_ this assay shows a decrease in filament formation for both LRRK2 wt and the C2025S mutant. Our results indicate that mutation of either of these two cysteine residues has strikingly different effects on the conformation of the AL. Like the D2017A mutation, the C2024S mutation potentially locks the kinase domain into a stable open and inactive conformation that is also kinase dead^18^.

Several LRRK2 mutations are directly linked to Parkinson’s disease (PD). Here we tested G2019S and I2020T, two of the most common PD-associated mutants, and these sites are also located in the AS and affect kinase activity. Two further PD mutations, where replacement with a cysteine residue has been reported are R1441C and Y1699C. Both are involved in a critical network allowing for crosstalk between the kinase domain and the GTPase domain^8,57,58^. Even though redox-based modulation varies between the pathogenic mutations, our data (Supplementary Fig. 4) shows a clear inhibition of the hyperactive G2019S, as well as I2020T and R1441C and Y1699C proteins in response to oxidizing agents. GaMD simulations suggest that the thiolation of C2024 specifically increases the time that the AL spends in close proximity to the COR-B:ROC interface where many PD mutations are located, including R1441C and Y1699C (Supplementary Fig. 1). Furthermore, CXL-1020 drastically inhibited the kinase activity of all tested pathogenic PD mutants. This points out the potential importance of analysing redox regulation of LRRK2 as a future strategy for developing selective kinase inhibitors and therapeutic agents.

Combining biochemical and cell-based data with GaMD simulations we propose a model of redox-dependent regulation of LRRK2 kinase activity by two adjacent cysteines, C2024 and C2025, in the AS. Redox-based regulation via differential action of each of those cysteines provides an extra layer of kinase regulation. In the inactive cryo-EM structure C2024 is part of the inhibitory helix, formed by the first 10 amino acids of the AS. GaMD simulations suggest that thiolation of C2024 influences the kinase core, in particular the HRD arginine (R1993), which is part of the catalytic loop. While the backbone of the HRD motif and the entire catalytic loop is very stable in all LRRK2 structures, the side chain of R1993 is one of the most dynamic residues in FL LRRK2 and in the LRRK2^RCKW^ structure. In LRRK2 the AL does not snap into a stable active conformation when C2024 is thiolated; however, when we looked at LRRK2^RCKW^ the AL comes closer to the ROC:COR-B interface when C2024 is thiolated, and the side chain of R1993 is restricted more to the AL region. We, therefore, propose that interaction between C2024^thiolate^ and R1993 stabilizes a more active-like conformation. In addition, in our GaMD simulations the side chain of R1933 interacts with Y2050, which is highly conserved in many kinases. In PKA, when the AL is phosphorylated on T197, Y215, which is the equivalent of Y2050 in LRRK2, interacts with the side chain of the HRD arginine, but also with the backbone amide of the phosphorylated residue (pT197). By this analogy we could also hypothesize that fully active LRRK2 is most likely phosphorylated on T2031 and that the reactivity of C2024 poises the AL for this final stage of assembly. Replacement of C2024 with serine would prevent this interaction between C2024^thiolate^ and R1993, which facilitates extension of the AS, as seen in the AlphaFold2 active LRRK2 structure. Thiolation of C2024 also allows M2027 to dock into the hydrophobic space between the COR-B:ROC interface. Thiolation of C2025, in contrast to C2024, appears to have minor effects on the positioning of the AL. While the two cysteines most likely contribute differently to redox regulation of LRRK2 kinase activity, both are required to achieve full catalytic activity.

## Methods

### Sequence alignment

A multiple sequence alignment of the Activation Segment of selected protein kinases was performed using CLUSTAL O (1.2.4). The following human protein sequences from UniProt were used: Homo sapiens; GenBank accession no. P17612 (PKACα), Q13976 (PKG1), O14965 (AURORA A), P31749 (AKT1), P12931 (SRC), Q38SD2 (LRRK1), Q5S007 (LRRK2).

### Cell culture, transfection and affinity purification of LRRK2

Human N-terminally FLAG-Strep-Strep (FSS-) -tagged LRRK2 constructs were expressed in HEK293T cells. Cultivation, transfection, expression and harvesting conditions of cells as well as affinity purification and storage of FSS-LRRK2 wt and mutants (R14411C, Y1699C, G2019S, I2020T, C2024S, C2024A, C2025S) were performed as recently described in Schmidt et al. ^35^ and Weng et al. 2023b^31^. Each fraction of FSS-LRRK2 purification was analysed by SDS-PAGE.

### Microfluidic mobility shift kinase assay

The functional protein concentration was determined via a microfluidic mobility shift assay (MMSA) using LRRKtide (RLGRDKYKTLRQIRQ-amide; GeneCust) as a peptide substrate. Therefore, LRRK2 was titrated with the high-affinity, ATP-competitive inhibitor, MLi-2 (Merck). First, 24 µL of Buffer A containing LRRK2 [104.2 nM], TrisHCl pH 7.5 [25 mM], NaCl [50 mM], MgCl_2_ [10 mM], DTT [1 mM], GDP [500 µM], BSA [0.5 mg/mL] were mixed with 1 µL of an MLi-2 (as 100% DMSO Stock) dilution series in a 384-well plate (Corning). Then, 10 µL of this reaction mixture was added to 10 µL Buffer B (25 mM) TrisHCl pH 7.5, 50 mM NaCl, 100 µM ATP, 1 mM DTT, 0.5 mg/mL BSA, 0.05% L31, 950 µM LRRKtide and 50 µM fluorescein-LRRKtide (Fluo-LRRKtide; GeneCust). Substrate conversion over time at 30 °C was monitored using a LabChip Reader Version 3.0. (PerkinElmer, Inc.). Data were analysed with GraphPad Prism 9.5.1. (GraphPad by Dotmatics Software). Percental conversion was plotted against time and slopes (percentage conversion/ min [%/min] were determined using a linear fit). Resulting conversion rates were plotted against the respective MLi-2 concentrations. Assuming a 1:1 binding of MLi-2, the functional protein concentration was determined by a linear fit.

This kinase assay was also used to determine the turnover numbers (k_cat_ [1/min]) of LRRK2 wt and mutants in the absence and presence of different reducing and oxidizing reagents. Therefore, LRRK2 [50 nM] was incubated in a kinase buffer (25 mM TrisHCl pH 7.5, 10 mM MgCl_2_, 1 mM ATP, 500 µM GDP, 0.5 mg/mL BSA and 0.05% L31) supplemented with 950 µM LRRKtide, 50 µM fluorescein-LRRKtide and either 1 mM Dithiothreitol (DTT; ROTH), 250 µM hydrogen peroxide (H_2_O_2_; Sigma-Aldrich), 250 µM 1-nitrosocyclohexalycetate (NCA) or CXL-1020 (AxonMEDCHEM) or the respective dilutions of named reducing and oxidizing agents. Substrate conversion was monitored as described above, while conversion rates were first converted into reaction velocities (v_0_ = micromoles per minute [µmol/min]) followed by conversion in turnover numbers (k_cat_). At least three independent measurements were performed with at least two independent protein preparations.

### In vitro autophosphorylation analysis and Rab8A phosphorylation assay with phospho-specific antibodies

Both, LRRK2 auto-phosphorylation analyses of pS1292 and Rab8A phosphorylation assays were recently described in Weng et al.^31^. Oxidation- and reduction-dependent phosphorylation of Rab8A was determined by adding 1 mM DTT, 250 µM H_2_O_2_, 250 µM NCA or 250 µM CXL-1020 to a kinase buffer (25 mM Tris pH 7.4, 50 mM NaCl, 10 mM MgCl_2_, 500 µM GDP, 1 mM DTT, 0.1 mg/mL bovine serum albumin (BSA)), supplemented with 1 mM ATP, 2.5 µM His_6_-Rab8A (6–175). Recombinantly expressed His_6_-Rab8A (6–175) was purified from *E.coli* BL21(DE3) RIL cells using Ni^2+^-NTA agarose (MACHEREY-NAGEL). For detection of Rab8A phosphorylation a primary antibody against pT72-Rab8A (1:5000; Abcam MJF-R20) was used as well as an anti-His6-Rab8A antibody (1:1.000; Abcam ab18184). LRRK2 auto-phosphorylation on S1292 was detected with an anti-pS1292 (1:1000; Abcam MJFR-19–7–8) antibody, in addition to an anti-Flag antibody (1:1000, Sigma-Aldrich (F1804)). As secondary antibodies the RDye® 800CW Donkey anti-Rabbit IgG Secondary Antibody (1:15000; LiCOR 926–32213) and IRDye® 680RD Goat anti-Mouse IgG Secondary Antibody (1:15000; LiCOR 926–68070) were used. Detection and quantification were performed using an Odysseys FC imaging system (LiCOR). All blots or gels derive from the same experiment and they were processed in parallel.

### Microtubule docking assay

LRRK2 full-length, wild-type, and mutants were examined for their ability to dock onto microtubules using a method previously described in Schmidt et al. 2019. HEK293T cells were seeded onto 6-well dishes containing poly-D-lysine–coated glass coverslips. Cells were transfected with the Lipofectamine 3000 reagent as per the manufacturer’s protocol using 2 μg of Flag‐Strep‐Strep‐(FSS)‐tagged LRRK2 DNA. After 48 h of transfection, the cells were treated with the LRRK2 inhibitor MLi-2 for 2 h before fixation and staining with anti-Flag antibody (Sigma-Aldrich F1804). Confocal imaging was performed with the Olympus Fluoview 1000 and Nikon AXR laser scanning confocal microscope using a 60X oil immersion objective lens with a numerical aperture of 1.42 and processed using the Fiji software package. Transfected cells were counted in the images, and the cells forming filaments were noted. Results were plotted as a percentage of transfected cells forming filaments in three independent experimental sets. Normal distribution was tested for using the Shapiro-Wilk test. Graphs were plotted using the GraphPad by Dotmatics Prism version 9.5.1. for Windows, GraphPad by Dotmatics Software, San Diego, California USA, www.graphpad.com.

### H_2_O_2_ treatment of cells

Following the method mentioned above, after 48 h of transfection, cells were incubated in plain DMEM for 30 min, followed by treatment with 250 μM H_2_O_2_ (Stock = 30% solution w/w) for 20 min. Subsequently, cells were treated with 100 nM MLi-2 for 2 h, following which the cells were fixed and stained.

### Gaussian accelerated Molecular Dynamics (GaMD) simulation

The simulation models for LRRK2^RCKW^ and FL LRRK2 were prepared using cryo-EM structures (PDB: 6VP6 and 7LHW, respectively). Missing loops in the protein structures were modeled using Modeller^59^, and the initial models were prepared by PyMOL 1.7.(Schrodinger, LLC; The PyMOL Molecular Graphics System). The full system was parameterized using LEaP in AMBER16. Cysteine residues were set to CYM to model the thiolated state. Hydrogens and counter ions were added, and the resulting models were solvated in a cubic box of TIP4P-EW water molecules and 150 mM KCl with a 10 Å buffer^60^. The systems were minimized through various steps, including hydrogen-only minimization, solvent minimization, ligand minimization, side-chain minimization, and all-atom minimization. For heating, the temperature was increased from 0 K to 100 K under constant volume over 50 ps using 2 fs time-steps and 5.0 kcal/mol•Å2 position restraints. Then, the temperature was raised from 100 K to 300 K under constant pressure over 200 ps, maintaining 5.0 kcal/mol/Å position restraints on the protein using the Langevin thermostat. For equilibration, a constant pressure simulation was run with a 10 Å non-bonded cut-off and 500 ps of restrained protein, followed by 300 ps of unrestrained equilibration. To enhance conformational sampling, Gaussian accelerated MD (GaMD) with GPU-enabled AMBER16 was used^61^. GaMD applies a Gaussian boost potential to accelerate transitions while allowing reweighting. Both dihedral and total boosts were applied. GaMD simulations involved 2 ns potential collection, 2 ns of GaMD with updating boosts, and 10 ns re-equilibration. For each system, a minimum of three independent 200 ns GaMD replicates were generated.

### Supplementary information

Supplementary Information

nr-reporting-summary

Figure/Video 5B. MD simulation revealed interactions stabilizing the inactive state of the Activation Loop in FL LRRK2

## Data Availability

The datasets used and/or analysed during the current study are available from the corresponding author.
