# Supplementary material for: RedOx regulation of LRRK2 kinase activity by active site cysteines
Source: NPJ Parkinsons Dis. 2024 Apr 3;10:75. doi: 10.1038/s41531-024-00683-5 (PMC10991482; doi:10.1038/s41531-024-00683-5)

# Supplementary Figures

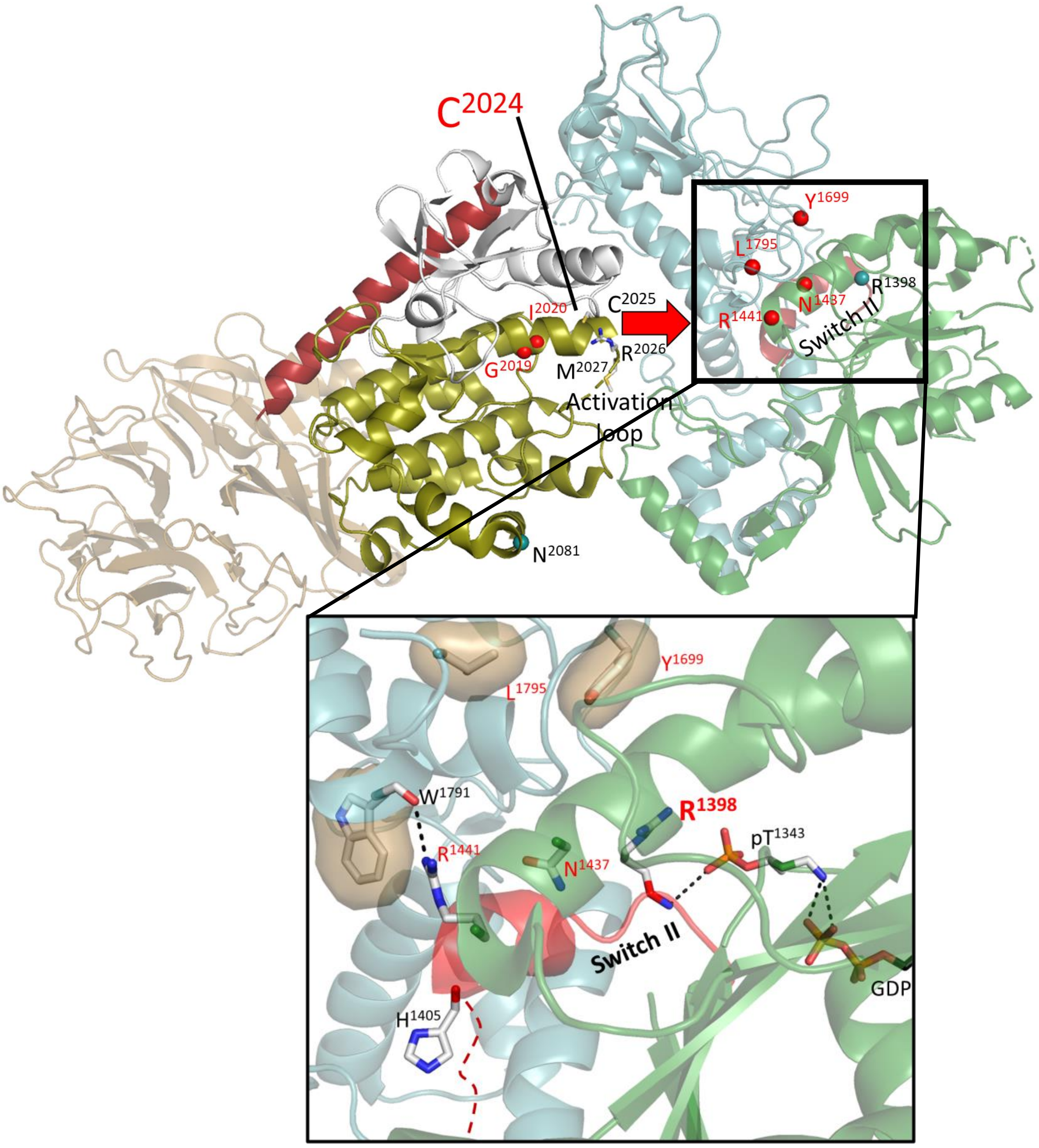


**Supplementary Figure 1. PD mutations in LRRK2**. Some PD mutations are shown in the inactive FL LRRK2 (PDB: 7LHW), C2024 and C2025 point towards the COR-B domain. The N- and C-Lobe of kinase domain are colored in white and tan, respectively, COR domains in teal, ROC in green, and WD40 in sand. The Switch II of ROC and C-tail helix (aa2500-2525) are highlighted in red. The two residues in the Activation Segment, R2026 and M2027 are also shown. Right, a close view of PD mutations in RCKW LRRK2 (PDB: 6VNO). The PD mutations are shown and labelled in red. Several key residues, pT1343, W1791 and GDP are also shown. H1405 from the Switch II (in red) is highlighted. The residues H1407 to Q1411 in Switch II are disordered, as shown in dash line.

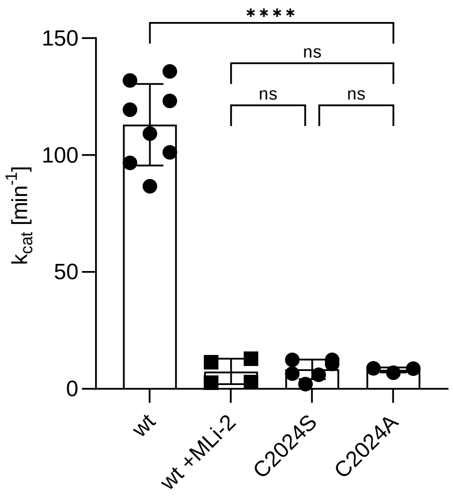


**Supplementary Figure 2. C2024 is critical for LRRK2 kinase activity.** Kinase activity towards LRRKtide was determined in a MMSA. The type I kinase inhibitor MLi-2 was used as a control in all assays. Data points represent the standard deviation (SD) of with three independent measurements based on a one-way ANOVA with a multiple comparison n.s.: P ≥ 0.05; ****:P < 0.0001.

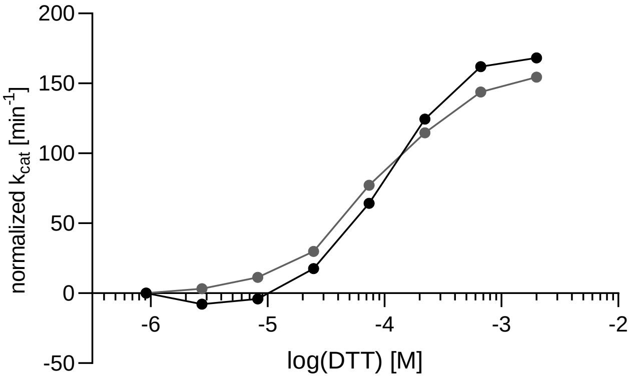


**Supplementary Figure 3. Dose-dependent activation of FL LRRK2 wt by DTT.** Kinase activity towards LRRKtide was determined in a MMSA, following incubation with different concentrations of DTT. A concentration-dependent activation of LRRK2 wt was measured, reaching maximal activity at 1 mM DTT. Normalized data points represent two independent measurements.

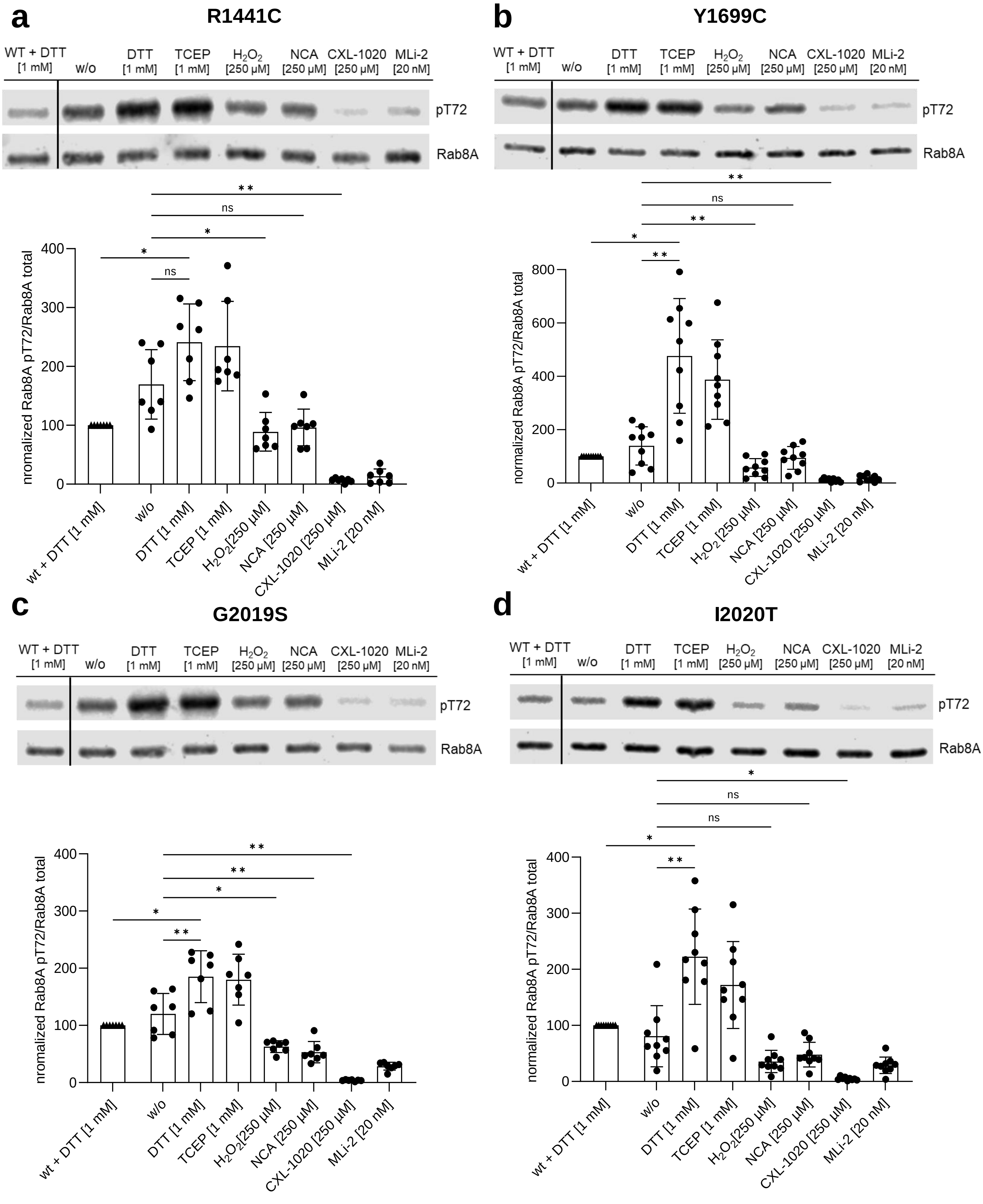


**Supplementary Figure 4. Redox-dependent regulation of LRRK2 pathogenic mutants. a-d** LRRK2 kinase activity determined by phosphorylation of Rab8A on T72 using a site-specific antibody (Abcam MJF-R20). Reduction (with DTT or TCEP; 1 mM each) of LRRK2 **a** R1441C, **b** Y1699C, c G2019S and **d** I2020T resulted in enhanced Rab8A phosphorylation, while oxidation (with $H_2O_2$, NCA, CXL-1020; 250 µM each) decreased kinase activity. MLi-2, an ATP-competitive inhibitor (type I) and buffer without any additional reductant or oxidant (w/o) were was used as controls. Not different scale in **b**. Representative Blots are shown, and data points represent the standard deviation (SD), of at least two protein preparations with at three independent measurements based on a one-way ANOVA with a multiple comparison n.s.: $P \geq 0.05$; **: $P < 0.01$; ***: $P < 0.001$; ****:$P < 0.0001$.

All blots were loaded in the same order as in the manuscript

# Full Scan Gel Images

## **Supplementary Figure 2**

**b** upper blot
pS1292 autophosphorylation
anti-pS1292 (green)
anti-LRRK2 (red)

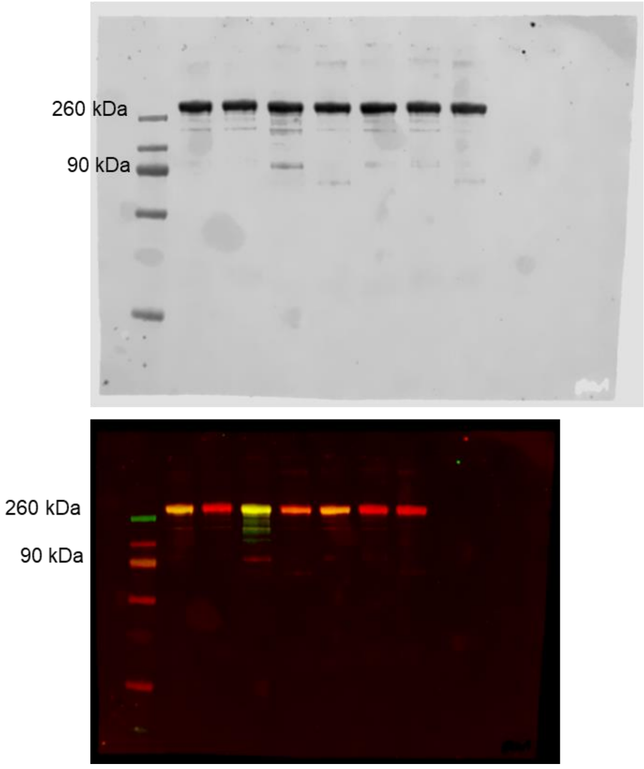


**b** lower blot
pT72 Rab8A phosphorylation
anti-pT72 (green)
anti-Rab8A (red)

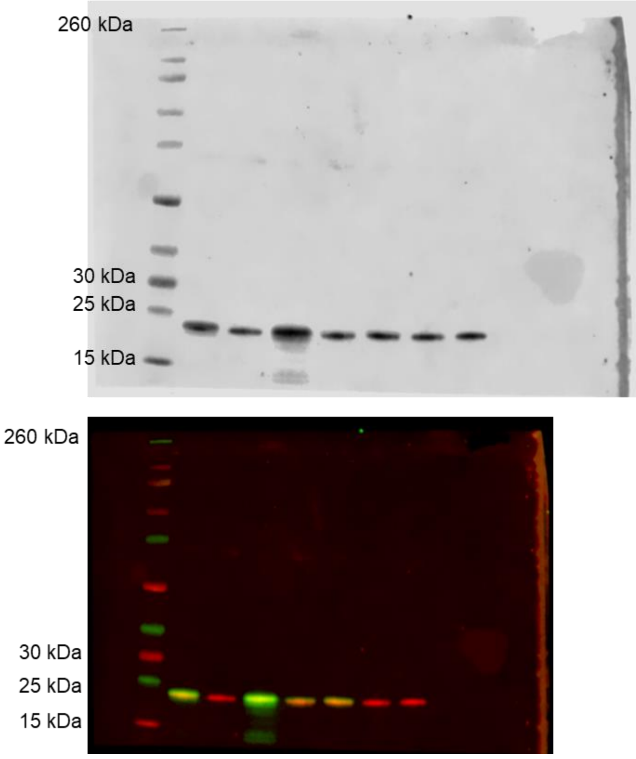

All blots were loaded in the same order as in the manuscript

# Full Scan Gel Images

## Supplementary Figure 4

**a** R1441C
pT72 Rab8A phosphorylation
anti-pT72 (green)
anti-Rab8A (red)

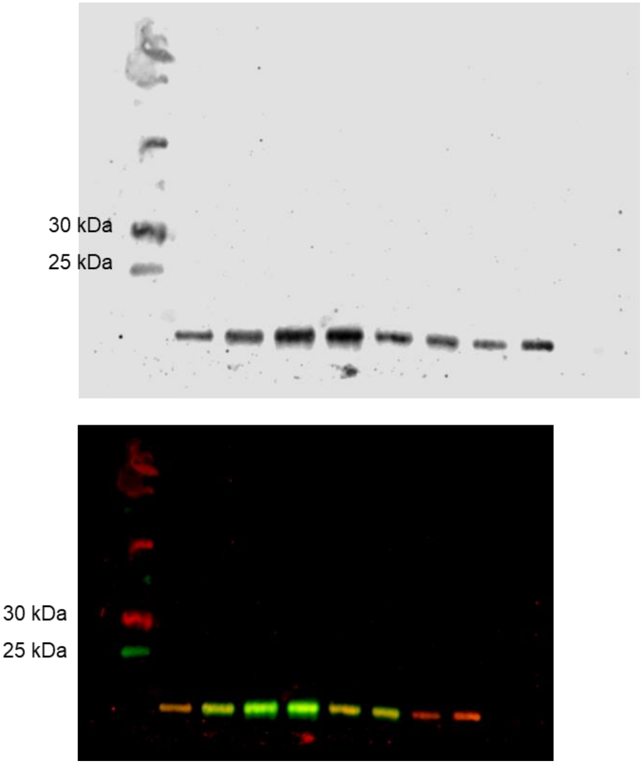


**b** Y1699C
pT72 Rab8A phosphorylation
anti-pT72 (green)
anti-Rab8A (red)

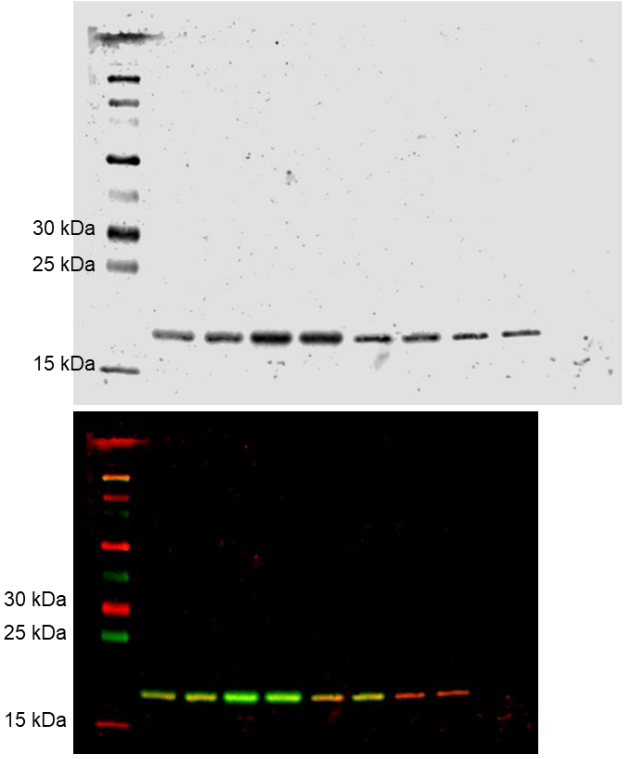


**c** G2019S
pT72 Rab8A phosphorylation
anti-pT72 (green)
anti-Rab8A (red)

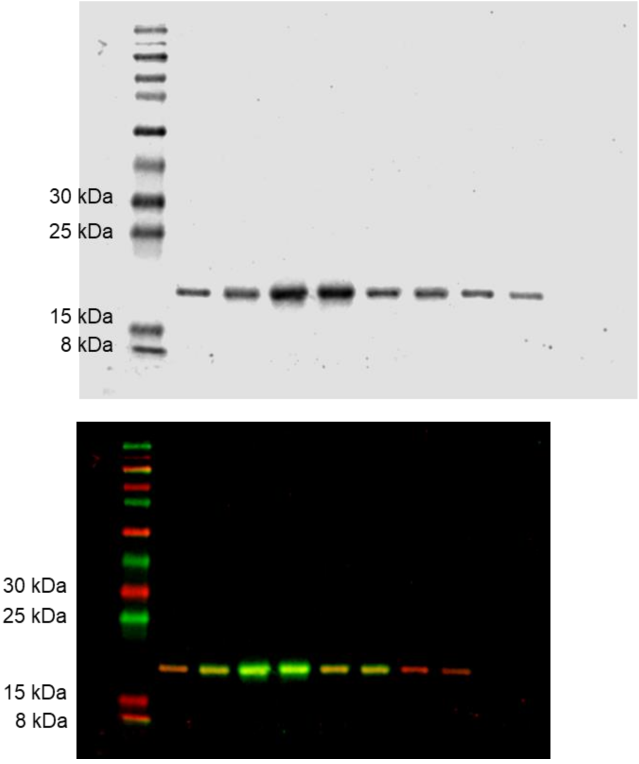


**d** I2020T
pT72 Rab8A phosphorylation
anti-pT72 (green)
anti-Rab8A (red)

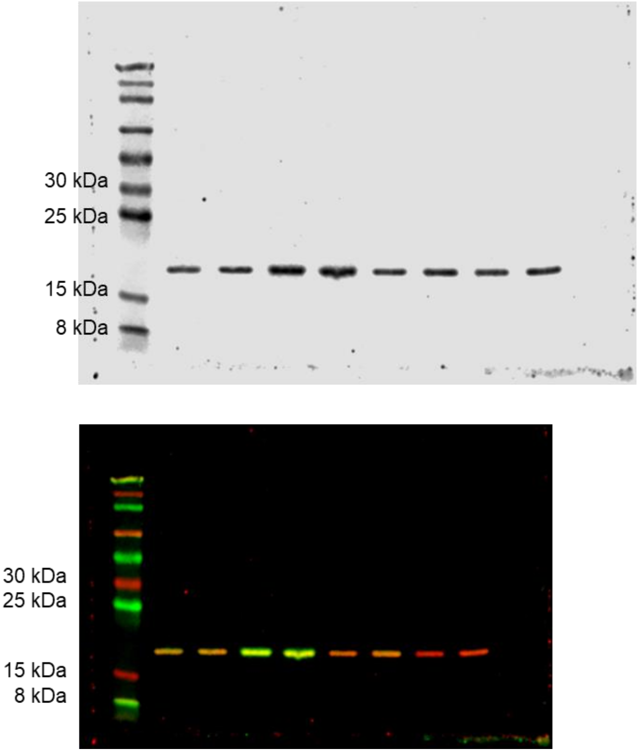

Supplement: Supplementary file 1 — Supplementary Information [file 41531_2024_683_MOESM1_ESM.pdf]
